# Critically ill metastatic cancer patients returning home after unplanned ICU stay: an observational, multicentre retrospective study

**DOI:** 10.1186/s13613-023-01170-5

**Published:** 2023-08-21

**Authors:** Frédéric Gonzalez, Rémi Starka, Laurent Ducros, Magali Bisbal, Laurent Chow-Chine, Luca Servan, Jean-Manuel de Guibert, Bruno Pastene, Marion Faucher, Antoine Sannini, Marc Leone, Djamel Mokart

**Affiliations:** 1 Polyvalent Intensive Care Unit, Department of Anesthesiology and Critical Care, Institut Paoli-Calmettes, 232 Boulevard Sainte Marguerite, 13009 Marseille Cedex 09, France; 2 Polyvalent Intensive Care Unit, Sainte Musse Hospital, Toulon, France; 3 Department of Anesthesiology and Intensive Care Unit, Nord Hospital, Assistance Publique Hôpitaux Universitaire de Marseille, Aix Marseille University, Marseille, France

**Keywords:** ICU, Metastatic cancer, Quality of life, Triage, 90-Day return home

## Abstract

**Background:**

Data about critically ill metastatic cancer patients functional outcome after unplanned admission to the ICU are scarce. The aim of this study was to assess factors associated with 90-day return home and 1-year survival in this population.

**Study design and methods:**

A multicenter retrospective study included all consecutive metastatic cancer patients admitted to the ICU for unplanned reason between 2017 and 2020.

**Results:**

Among 253 included metastatic cancer patients, mainly with lung cancer, 94 patients (37.2%) could return home on day 90. One-year survival rate was 28.5%. Performance status 0 or 1 (OR, 2.18; 95% CI 1.21–3.93; *P* = 0.010), no malnutrition (OR, 2.90; 95% CI 1.61–5.24; *P* < 0.001), female gender (OR, 2.39; 95% CI 1.33–4.29; *P* = 0.004), recent chemotherapy (OR, 2.62; 95% CI 1.40–4.90; *P* = 0.003), SOFA score ≤ 5 on admission (OR, 2.62; 95% CI 1.41–4.90; *P* = 0.002) were significantly predictive for 90-day return home. Malnutrition (HR, 1.66; 95% CI 1.18–2.22; *P* = 0.003), acute respiratory failure (ARF) as reason for admission (HR, 1.40; 95% CI 1.10–1.95; *P* = 0.043), SAPS II on admission (HR, 1.03; 95% CI 1.02–1.05; *P* < 0.001) and decisions to forgo life-sustaining therapies (DFLST) (HR, 2.80; 95% CI 2.04–3.84; *P* < 0.001) were independently associated with 1-year mortality.

**Conclusions:**

More than one out of three metastatic cancer patients could return home within 3 months after an unplanned admission to the ICU. Previous performance and nutritional status, ongoing specific treatment and low severity of the acute illness were found to be predictive for return home. Such encouraging findings should help change the dismal perception of critically ill metastatic cancer patients.

## Introduction

Cancer is the second most common cause of death in the world [1]. Continuous improvements in early diagnosis, oncologic treatment and supportive care led to better outcome [2]. New therapeutic approaches targeting actionable oncogenic mutations in tumour cells or their immune microenvironment may provide a prolonged survival with acceptable quality of life to patients formerly considered refractory [3]. These advances result in a rising number of patients with active cancer at risk of developing life-threatening complications [4], and a recent observational study found that 5% of cancer patients experience a critical illness resulting in intensive care unit (ICU) admission within 2 years after cancer diagnosis [5]. In a worldwide survey including 10069 patients admitted to the ICU from 730 centres in 84 countries, 888 (8.8%) had solid non-metastatic cancer and 332 (3.3%) had metastatic cancer [6]. Advanced or metastatic stage of underlying malignancy is often reported as worsening short- or medium-term prognosis in critically ill cancer patients, and oncologists and intensivists are often reluctant to propose or admit these patients to the ICU [7]. Data on outcome of critically ill metastatic cancer patients are scarce and studies in the field often assess survival rate [4, 8–12]. Few studies evaluated the clinical evolution after ICU discharge, in terms of functional status, quality of life and ability to receive further specific treatment [13, 14].

The aim of our study was to determine the factors associated with survival of metastatic cancer patients admitted to the ICU, particularly those associated with 90-day return home, a pragmatic endpoint aligned with patients’ individual goals and needs [15].

## Patients and methods

### Main objective

The primary objective of this study was to determine the predictive factors for return home after an unplanned ICU stay among metastatic solid cancer patients. The secondary objective was to assess factors associated with 1-year survival in this population.

### Study design, settings and participants

We performed a multicentre retrospective study including all consecutive adult patients (age ≥ 18 years) with metastatic solid tumour requiring unplanned admission to three ICUs between January 1, 2017 and December 31, 2020. Non-inclusion criteria were: admission to secure a procedure, planned admission following elective surgery and patients with testicle cancer, as the course and management of this subtype of metastatic cancer is very specific. For patients with multiple ICU admissions, only the first qualifying ICU stay was considered.

The study was conducted at the Paoli-Calmettes Institute, a comprehensive cancer center, Nord Teaching Hospital (Assistance-Publique Hôpitaux de Marseille), an academic center, both in Marseille (France), and at the Sainte Musse Hospital in Toulon (France), a general hospital.

All management decisions were independently made by the attending physicians according to standard practices. According to French regulations, this study was approved by our local Institutional Review Board (n°2020–067), which waived the need for signed consent.

### Data collection

The following variables were collected on admission: age, gender, medical background, comorbidities, type of solid tumour, primary tumour site, metastases type and number of sites, main admission reason, previous anticancer treatments (surgery, radiotherapy, cytostatic chemotherapy, targeted therapy, immunotherapy) and number of lines (i.e., number of lines of specific cancer therapy before ICU admission, including or not chemotherapy). Two study investigators reviewed main diagnoses.

The severity of the illness was evaluated using the Sequential Organ Failure Assessment (SOFA) score and the Simplified Acute Physiology Score II (SAPS II) at admission. Comorbidities were determined with the Charlson Comorbidity Index (CCI). Eastern cooperative oncology group performance status (ECOG-PS) 1 month before ICU admission was recorded. Malnutrition was defined as body mass index (BMI) < 18.5 kg/m^2^, weight loss ≥ 5% in 1 month or ≥ 10% in 6 months before ICU admission, or albumin < 30 g/L on ICU admission. Clinical and laboratory data at ICU admission were collected, including neutropenia and its duration (absolute neutrophil count of < 0.5 × 10^9^/L), as well as organ failures during ICU stay. The Oncoscore was calculated [14]. All microbiological documentations were recorded.

The ICU and hospital length of stay and the time between hospital and ICU admission were calculated. The ICU interventions were defined by the use of mechanical ventilation (MV), including non-invasive ventilation (NIV) and invasive mechanical ventilation (IMV), the use of vasopressors, renal replacement therapy (RRT) and oncologic treatments.

During the ICU stay, life-supporting interventions, anti-infectious agents, prophylactic treatments, and diagnostic procedures were administered at the discretion of the attending intensivist, following best clinical practice and guidelines. Chemotherapy, corticosteroids, hematopoietic growth factors, immunosuppressive drugs and other cancer-related treatments were prescribed by the oncologist in charge in accordance with institutional guidelines. Decisions on ICU discharge were left at the discretion of the attending intensivist, and patients were discharged from ICU without any non-hematological organ failure.

Decisions to forgo life-sustaining therapies (DFLST) before admission and during ICU stay were recorded. Progression free survival and anti-neoplastic therapy after ICU discharge were reported.

### Study outcomes

The number of patients returned home on day 90, as well as the ICU, hospital, and 1-year mortality rates were recorded.

### Statistical analysis

All data are presented as rates (percentages) for qualitative variables and as medians (25–75th percentiles) for the categorical variables. Patients’ features during the first 90 days after ICU admission were compared across two groups of patients: patients returned home on day 90 versus patients not returned home on day 90. Since we knew the immediate location of all patients discharged alive from the hospital, we classified the 14 patients lost to follow-up 90 days after admission to the ICU in the "no 90-day return home" group. Comparisons between the two groups for continuous variables were made using the Mann–Whitney test. Comparisons between the two groups for categorical variables were made using the Pearson Chi-square test or Fisher exact test. All *P* values < 0.05 were considered to be statistically significant. We performed logistic regression analyses to identify the independent variables associated with 90-day return home, measured by odds ratio (OR) with a 95% confidence interval (95% CI). Variables yielding a P lower than 0.10 in the bivariate analyses were entered a backward stepwise logistic regression model. One-year survival was calculated from the day of ICU admission until death from any cause (event) or from the last follow-up before death (censored), the follow-up period was censored at 1 year. Correlations between patient characteristics and 1-year mortality were assessed using univariate and multivariate Cox regression models, measured by the estimated hazard ratio (HR) with a 95% confidence interval (95% CI). All statistical tests were two-sided. The required significance level was set at a P value less than 0.05. The Cox proportional hazard model used in the multivariate approach included variables for which a P value less than 0.10 was observed in the univariate analysis as well as clinically relevant variables. A backward stepwise selection procedure (threshold of 0.05) was used to retain the final model. The Cox proportional hazards assumptions were tested using goodness-of-fit tests based on the cumulative sums of the martingale residuals [16]. The variable of interest was 1-year mortality, and results were expressed as HR and 95% CIs.

Statistical tests were conducted using the R software (R Core Team, 2020).

## Results

**Fig. 1:**
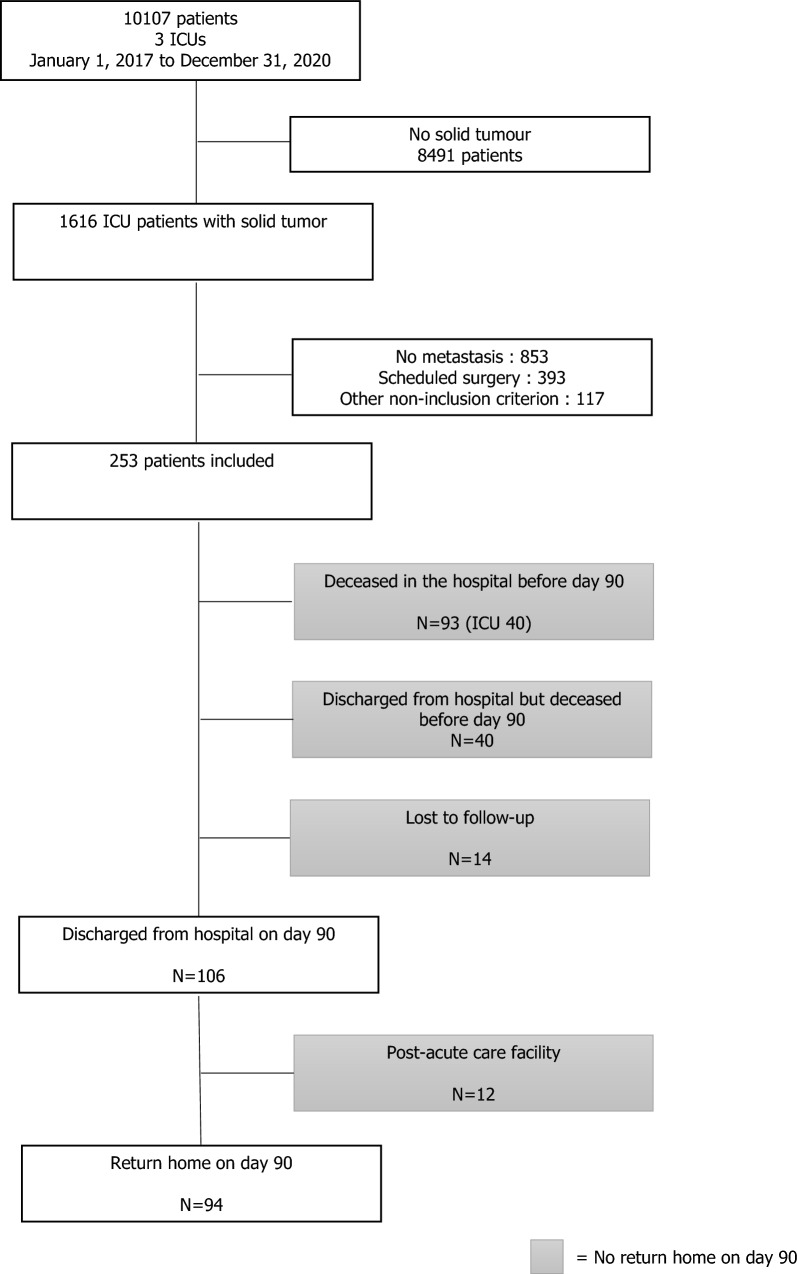
Flow chart

During the study period, 253 patients, aged of 65 years [56–72] with a 1:1 sex ratio , were included (Fig. 1). Their features are presented in Table 1. The CCI was 10 [8–11]. The most frequent primary tumour sites were lung for 95 (37.5%) patients, breast (16.2%) and colorectal (9.1%). The median number of metastatic sites was 2 [1–3], mainly lung/pleura (49%), bone (40.7%) and liver (38.3%). One hundred and one (39.9%) patients had an ECOG-PS of 0 or 1 one month before ICU admission. Before ICU admission, the median number of administered treatment lines was 2 [0–2], whereas 68 (23.9%) patients did not receive any treatment. Malnutrition was identified in 137 (53.5%) patients.

**Table 1:** Baseline characteristics of the study population according to 90-day return home status

	Total (*n* = 253)	90-day return home (*n* = 94)	No 90-day return home (*n* = 159)	*p*
Age, y	65 [56–72]	63 [54–69]	65 [58–74]	0.03
Male gender	126 (49.8)	33 (35.1)	93 (58.5)	0.001
Charlson Comorbidity Index	10 [8–11]	9 [8–10]	10 [8–11]	0.045
Performance Status 1 month before ICU admission				
0–1	101 (40)	52 (55)	49 (32)	< 0.001
2–3–4	150 (59)	41 (44)	109 (71)	< 0.001
Malnutrition	137 (54)	33 (35)	104 (65)	< 0.001
Albumin on day 1	28 [23–31]	30 [24–32]	27 [22–29]	0.002
Primary tumour site				
Lung, non-small cell	82 (33)	25 (27)	57 (36)	0.17
Breast	41 (16)	22 (24)	19 (12)	0.027
Colorectal	23 (9)	10 (10)	13 (8)	0.67
Kidney	12 (5)	2 (2)	10 (6)	0.23
Lung, small cell	13 (5)	6 (7)	7 (4)	0.69
Prostate	10 (4)	4 (4)	6 (4)	1.00
Stomach	9 (4)	2 (2)	7 (4)	0.55
Pancreas	9 (4)	5 (5)	4 (3)	0.42
Bladder	9 (3)	3 (3)	6 (4)	1.00
Hepatobiliary	8 (3)	0 (0)	8 (5)	0.30
Other	37 (14)			
Metastatic site				
Lung/pleura	124 (49)	48 (51)	76 (50)	1.00
Bone	103 (41)	38 (40)	65 (41)	1.00
Liver	97 (38)	32 (34)	65 (41)	0.34
Brain	44 (17)	18 (19)	26 (16)	0.69
Peritoneum	39 (15)	19 (20)	20 (13)	0.15
Adrenal gland	28 (11)	8 (9)	20 (13)	0.43
Other	23 (9)			
Metastatic sites, n	2 [1–3]	2 [1–3]	2 [1–3]	0.39
LDH on day 1 (UI/L)	321 [219–478]	315 [190–434]	329 [232–619]	0.165
Antineoplastic therapy before ICU admission				
Cytostatic chemotherapy	148 (59)	67 (71)	81 (51)	0.002
Endocrine therapy	35 (14)	16 (17)	19 (12)	0.35
Targeted therapy/immunotherapy	89 (35)	37 (40)	52 (33)	0.35
Radiotherapy	70 (27)	31 (33)	39 (25)	0.17
No previous treatment	68 (27)	13 (14)	55 (35)	0.001
Lines of antineoplastic therapy before admission, n	2 [0–2]	1 [1–3]	1.00 [0–2]	< 0.001

Quantitative data are reported as median [IQR] and categorical data as n (%)

*IQR* interquartile range, *ICU* Intensive Care Unit, *LDH* lactate dehydrogenase

The ICU stay characteristics are presented in Table 2. Reasons for ICU admission were mainly acute respiratory failure in 149 (58.9%) patients and septic shock in 53 (20.9%). Infection represented 46.2% of final etiological diagnoses. Tumour-related reason for ICU admission was present in 92 (36.4) patients, and anti-neoplastic drug side effect was reported in 22 (8.7%) patients.

**Table 2:** ICU stay descriptive data according to 90-day return home status

	Total (*n* = 253)	90-Day return home (*n* = 94)	No 90-day return home (*n* = 159)	*p*
Reason for ICU admission				
Acute respiratory failure	149 (58)	49 (52)	100 (63)	0.12
Septic shock	53 (21)	13(14)	40 (25)	0.048
Coma	41 (16)	15 (16)	26 (16)	1.00
Acute renal failure	31 (12)	11 (12)	20 (13)	0.994
Life-threatening metabolic complication	20 (8)	13 (14)	7 (4)	0.015
Hypercapnic respiratory failure	18 (7)	7 (7)	11 (7)	1.00
Severe bleeding	13 (5)	5 (5)	8 (5)	1.00
Main etiological diagnosis				
Infection	117 (46)	41 (44)	76 (50)	0.519
Pneumonitis	71 (28)	50 (53)	21 (13)	
Intra-abdominal	30 (12)	20 (21)	10 (6)	
Urinary tract	11 (4)	5 (5)	6 (4)	
Specific, related to the underlying cancer	92 (36)	32 (43)	60 (38)	0.65
Pleural effusion	38 (15)	15 (16)	23 (15)	0.89
Anti-neoplastic drug side effect	22 (9)	11 (12)	11 (7)	0.28
Cardiogenic pulmonary oedema	14 (6)	4 (4)	10 (6)	0.69
Aspiration pneumonitis	10 (4)	0 (0)	10 (6)	0.032
Pulmonary embolism	9 (4)	3 (3)	6 (4)	1.00
Pericardial effusion, cardiac tamponade	6 (2)	3 (3)	3 (2)	0.82
Organ failure supports				
Vasopressors	105 (41.5)	29 (31)	76 (50)	0.008
Mechanical ventilation	60 (23.7)	15 (16)	45 (29)	0.026
Renal replacement therapy	7 (3)	0 (0)	7 (5)	0.039
SAPS II	45 [38–53]	42 [36–47]	48 [41–56]	< 0.001
SOFA score on day 1	5 [3–8]	4 [3–6]	6 [4–9]	< 0.001
SOFA score > 5 on day 1	106 (42)	25 (27)	81 (51)	< 0.001
∆ SOFA day 1–day 3	3 [0–3]	2 [1–4]	2 [0.5–3]	0.045
ONCOSCORE	4 [3–6]	4 [3–5]	4 [3–6]	0.001
Severe neutropenia	17 (7)	9 (10)	8 (5)	0.25
Thrombocytopenia	67 (26)	25 (27)	42 (26)	1.00
Time since first symptoms, d	2 [1–6]	2 [1–5]	2 [1–6]	0.66
ICU LOS, d	3 [3–7]	4 [3–7]	4 [3–7]	0.78
DFLST before ICU admission	5 (2)	1 (1)	4 (2.5)	0.74
DFLST during ICU stay	90 (36)	15 (16)	75 (47)	< 0.001

Quantitative data are reported as median [IQR] and categorical data as n (%)

*IQR* interquartile range, *ICU* Intensive Care Unit, *SAPS II* Simplified Acute Physiology Score II, *SOFA* Sequential Organ Failure Assessment, *LOS* Length of Stay, *DFLST* Decisions to forgo life-sustaining therapies

Vasopressors and mechanical ventilation were required in 105 (41.5%) and 60 (23.7%) patients, respectively. The SAPS II, the SOFA score on day 1 were 45 [38–53] and 5 [3–8], respectively, while Oncoscore was 4 [3–6]. DFLST during the ICU stay were reported for 90 (35.6%) patients.

### Outcome

#### 90-Day return home

On day 90, 94 (37.2%) patients returned home. The ICU, hospital, 90-day and 1-year mortality rates were 15.8%, 36.8%, 52.6% and 71.5%, respectively. Thirty-two (12.6%) patients, who were discharged alive from hospital, were lost to follow-up at 1 year.

Among the 159 (62.8%) patients who did not return home on day 90, 93 (36.8%) died in the hospital, 40 (15.8%) died within 3 months, after hospital discharge, 14 (5.5%) were lost to follow-up and 12 (4.7%) were admitted to post-acute care facilities.

##### Univariate analysis

Factors associated with 90-day return home and 1-year mortality are presented in Tables 1, 2 and 3**.**

**Table 3:** Univariate analysis of factors associated with 1-year mortality

	HR	95% CI	*p*
Age, per year	1.01	1.00–1.03	0.059
Female gender	0.67	0.50–0.90	0.008
Charlson Comorbidity Index	1.05	0.96–1.13	0.290
Performance Status 1 month before ICU admission			
0–1	0.46	0.34–0.63	0.001
Malnutrition	2.08	1.47–2.70	0.001
Albumin on day 1	0.97	0.95–1.00	0.079
Primary tumour site			
Lung, non-small cell	1.26	0.93–1.71	0.14
Lung, small cell	1.14	0.62–2.10	0.67
Breast	0.55	0.35–0.87	0.010
Colorectal	1.19	0.73–1.93	0.49
Kidney	1.776	0.961–3.281	0.067
Prostate	0.49	0.20–1.18	0.113
Stomach	1.545	0.725–3.291	0.260
Pancreas	1.333	0.425–4.176	0.622
Bladder	1.065	0.5–2.268	0.87
Metastatic site			
Lung/pleura	0.97	0.72–1.32	0.86
Bone	0.93	0.69–1.25	0.62
Liver	1.42	1.06–1.91	0.019
Adrenal gland	1.32	0.86–2.03	0.20
Metastatic sites, n	1.07	0.94–1.22	0.30
LDH on day 1 (UI/L)	1.002	1–1.004	0.024
Antineoplastic therapy before ICU admission			
Cytostatic chemotherapy	0,55	0.40–0.74	< 0.001
Hormone therapy	0.58	0.37–0.93	0.024
Targeted therapy/immunotherapy	0.88	0.65–1.19	0.403
Radiotherapy	0.55	0.38–0.79	0.001
No previous treatment	2.13	1.55–2.92	< 0.001
Lines of antineoplastic therapy before admission	0.83	0.74–0.93	0.002
Reason for ICU admission			
Acute respiratory failure	1.42	1.05–1.91	0.024
Septic shock	1.34	0.94–1.89	0.102
Coma	1.07	0.72–1.59	0.75
Acute renal failure	1.11	0.71–1.74	0.64
Life-threatening metabolic complication	0.63	0.35–1.13	0.119
Hypercapnic respiratory failure	1.12	0.64–1.97	0.696
Severe bleeding	0.80	0.39–1.63	0.538
Multiple organ failure	4.30	2.21–8.82	< 0.001
Etiological diagnosis			
Infection	0.93	0.69–1.23	0.600
Specific, related to the underlying cancer	1.31	0.97–1.76	0.074
Pleural effusion	1.01	0.68–1.52	0.952
Anti-neoplastic drug side effect	0.76	0.44–1.32	0.092
Cardiogenic pulmonary oedema/cardiac failure	1.34	0.73–2.46	0.3511
Aspiration pneumonitis	1.38	0.68–2.80	0.375
Pulmonary embolism	1.37	0.64–2.91	0.421
Pericardial effusion, cardiac tamponade	0.67	0.25–1.79	0.421
Organ failure supports			
Vasopressors	1.35	1.01–1.81	0.046
Mechanical ventilation	1.47	1.05–2.05	0.024
Renal replacement therapy	2.52	1.18–5.41	0.017
SAPS II	1.03	1.02–1.04	0.001
SOFA score on day 1	1.09	1.05–1.14	0.001
SOFA score > 5 on day 1	1.86	1.39–2.49	< 0.001
∆ SOFA d1–d3	0.89	0.85–0.94	< 0.001
ONCOSCORE	1.11	1.04–1.19	0.003
Severe neutropenia on day 1	0.67	0.35–1.26	0.215
Thrombocytopenia on day 1	0.95	0.68–1.34	0.776
Time since first symptoms, d	1.00	0.98–1.01	0.551
ICU LOS, d	0.99	0.96–1.01	0.292
DFLST before ICU admission	0.91	0.29–2.84	0.868
DFLST during ICU stay	3.67	2.73–4.99	< 0.001

*HR* hazard ratio, *95% CI* 95% confidence interval, *ICU* Intensive Care Unit, *LDH* lactate dehydrogenase, *SAPS II* Simplified Acute Physiology Score II, *SOFA* Sequential Organ Failure Assessment, *LOS* Length of Stay, *DFLST* Decisions to forgo life-sustaining therapies

Young age, female gender, low CCI, ECOG-PS at 0 or 1, good nutritional status and breast primitive tumour were associated with 90-day return home. Moreover, on-going chemotherapy for the underlying malignancy before ICU stay favoured 90-day return home. Conversely, increased ICU severity scores (i.e., SOFA score and SAPS II) were predictive for reduced rates of 90-day return home.

##### Multivariate analysis

In multivariate analysis, five factors were independently associated with 90-day return home (Table 4). ECOG-PS 0 or 1 (OR, 2.18; 95% CI 1.21–3.93; *P* = 0.010), lack of malnutrition (OR, 2.90; 95% CI 1.61–5.24; *P* < 0.001), female gender (OR, 2.39; 95% CI 1.33–4.29; *P* = 0.004), recent chemotherapy (i.e., within the previous 3 months) (OR, 2.62; 95% CI 1.40–4.90; *P* = 0.003), SOFA score ≤ 5 on ICU admission (OR, 2.62; 95% CI 1.41–4.90; *P* = 0.002) were significantly predictive of 90-day return home.

**Table 4:** Multivariate analysis for factors associated with 90-day return home

	Odds ratio	95% CI	*p*
Female gender	2.39	1.33–4.29	0.004
Performance status 0 or 1	2.18	1.21–3.93	0.010
Absence of malnutrition	2.90	1.61–5.24	< 0.001
Chemotherapy before ICU admission	2.62	1.40–4.90	0.003
SOFA on day 1 ≤ 5	2.62	1.41–4.90	0.002

*95% CI* 95% confidence interval, *ICU* Intensive Care Unit, *SOFA* Sequential Organ Failure Assessment

#### 1-year mortality

The results of the univariate analysis for 1-year mortality are shown in Table 3. In multivariate analysis, malnutrition (HR, 1.66; 95% CI 1.18–2.22; *P* = 0.003), acute respiratory failure (ARF) as reason for admission (HR, 1.40; 95% CI 1.10–1.95; *P* = 0.043), SAPS II on admission (HR, 1.03; 95% CI 1.02–1.05; *P* < 0.001) and DFLST (HR, 2.80; 95% CI 2.04–3.84; *P* < 0.001) were independently associated with 1-year mortality. In contrast, recent chemotherapy (HR, 0.71; 95% CI 0.52–0.96; *P* = 0.029) and hormone therapy (HR, 0.57; 95% CI 0.35–0.93; *P* = 0.024) were associated with 1-year survival (Table 5).

**Table 5:** Multivariate analysis for factors associated with 1-year mortality

	HR	95% CI	*p*
Malnutrition	1.61	1.18–2.22	0.003
Cytostatic chemotherapy before ICU admission	0.71	0.52–0.96	0.029
Hormone therapy before ICU admission	0.57	0.35–0.93	0.024
Acute respiratory failure (as reason for ICU admission)	1.40	1.10–1.95	0.043
SAPS II	1.03	1.02–1.05	< 0.001

HR hazard ratio, *95% CI* 95% confidence interval, *SAPS II* Simplified Acute Physiology Score II

## Discussion

Patients with metastatic solid malignancies are often denied access to the ICU because of predicted poor outcome. Indeed, previous studies reported high mortality rates in similar patients. Recent advances in oncological treatment, mainly targeted therapies and immunotherapy, and specific supportive care led to improved survival in critically ill patients with metastatic solid malignancies. Yet, only few studies assessed the functional status or the quality of life of these patients in the era of new therapeutic strategies [17]. We report here the results of our multicentre retrospective study in this specific population, using a pragmatic outcome endpoint, i.e., 90-day return home.

In our study, 94 out of 253 critically ill metastatic cancer patients returned home within 90 days after an unplanned ICU stay. One-year mortality was 71.5%. ICU-mortality and hospital mortality were 16% and 37%, respectively. Considering that we included only metastatic patients with unplanned ICU admission, one could expect higher mortality rates. Yet, our results are consistent with most recent studies, as survival in this population constantly improved during the two past decades [10, 18]. Indeed, in a similar setting, Ha et al. reported in 2017 an ICU-mortality and hospital mortality of 16% and 37%, respectively [8], while Vigneron et al. mentioned a 1-year mortality of 78.5% among critically ill patients with mainly advanced or metastatic cancer. In this study, ICU-mortality and hospital mortality were 24.8% and 44.3%, respectively [4].

In our study, ECOG-PS from 0 to 1 and the lack of malnutrition favoured 90-day return home (Table 4), while malnutrition was independently associated with 1-year mortality (HR 1.61, 95% CI [1.18–2.22], *P* = 0.003) (Table 5). Our findings are consistent with recent studies, highlighting the dominance of previous physiological condition, such as ECOG-PS, on underlying malignancy’s characteristics for short- and long-term survival [9, 19, 20]. Hospital mortality seems more associated with acute organ failure during the ICU stay than with cancer features [8, 12, 13, 21]. Interaction and communication between oncologists and intensivists are needed to find the balance point, avoiding both futile treatments and loss of chance.

As return home after ICU stay has been poorly investigated in this specific subgroup of patients, we cannot compare our results to previous findings. However, 37.2% of critically ill metastatic cancer patients returning home after an ICU stay should prompt us to revise misconceptions about actual outcome and consider broader admission criteria or time limited trials in this specific population. The ICU and hospital mortality are solid endpoints, but view from patients and their relatives, short-term return home, reflecting an acceptable quality of life may be a critical achievement. Indeed, often aware of the incurable nature of the disease, the patients rarely request survival at any cost.

Considering this innovative endpoint, we found questioning results on factors associated with return home in this very specific population. Indeed, unlike determinants of ICU-, hospital and 1-year survival, the primary site of cancer did not seem to influence return home. Our cohort included 82 (33%) patients with non-small cell lung cancer, known as high-risk malignancy [14, 22]. Nevertheless, this type of cancer did not result in higher mortality rates than others. In addition, it did not jeopardized return home. Yet, as female gender is associated with return home, one could hypothesize that breast cancer may influence this result in larger studies.

Moreover, cancer-related complications per se as main diagnosis did not influence the rate of return home in our cohort. However, previous studies underlined cancer-related complications as predictive of short- and long-term mortality [23, 24]. We hypothesized that, due to a better knowledge of prognostic factors and new fast-acting targeted therapies available, the patients with metastatic solid malignancy admitted to our ICUs were carefully selected. Another uncommon result of our study was that recent chemotherapy significantly favours return home in multivariable analysis (OR 2.62, 95% CI [1.40–4.90], *P* = 0.003). Such result was previously reported in only one study [13]. Actually, it probably reflects good general functional status of patients able to receive active treatment and might be a sign of previous triage decisions.

Previous triage decisions, in the oncology ward or emergency department, are critical for this population, although they could not be assessed in our study. We think that the potential reversibility of acute life-threatening cancer-related complications may have guided the admission decision-making process, leading to better outcome.

As expected, DFLST were an independent risk factor for 1-year mortality, in accordance with previous recent studies [4, 22]. As DFLST process is complex with many intricate variables influencing the decision, it is likely that determining factors may have not been recorded nor included in the analysis. Of note, DFLST during ICU stay did not systematically lead to death of the patient, since 16% of those returning home had DFLST during their ICU stay. This highlights the difficulty of triage decisions in this setting.

Yet, we cannot exclude that self-fulfilling prophecies played a role in the association between DFLST and mortality. In fact, in this very vulnerable population with a presumed poor outcomes, physicians might be prompt to withdraw or withhold life-sustaining therapies, leading to high-mortality rates and thus confirming the presumed poor outcomes [25]. This circular reasoning has been reported in several studies, mainly concerning patients with intracerebral haemorrhage, traumatic brain injury or cardiac arrest survivors [26, 27]. One could hypothesize that self-fulfilling prophecy might occur in critically ill metastatic cancer patients, as ICU admission and full-code resuscitation is often considered as futile by intensivists [7, 28].

Our results argue for a paradigm shift considering ICU admission for critically ill patients with metastatic cancer. This change should be in line with new admission criteria, based on the stage of the underlying neoplasia and the ICU stay expectations, sharing this perspective with the patients and/or her/his relatives. We believe that return home is perhaps a more meaningful endpoint than the 28-day or 1-year mortality rates. The patient and relatives may also decide if the process of end-of-life at home is a preferred option [29–31]. With this in mind, future studies evaluating outcome factors and ICU admission criteria of critically ill patients with advanced-stage/metastatic cancer should investigate new endpoints, taking into account quality of life, functional status and patients’ wishes and preferences, not only crude mortality rates, as developed in a study based on a large database, assessing the number of day at home as future endpoint [32].

The strength of our study relies on its multicentre design, including a large number of critically ill metastatic patients with solid cancer, included on a short period, considering therapeutic innovations, such as targeted therapies and immunotherapy. Indeed, 89 (35%) patients had received targeted therapy or immunotherapy before ICU admission. Moreover, we chose an unusual but pragmatic outcome endpoint (i.e., 90-day return home) and our results suggested that ICU admission for selected patients was not futile in this specific population. In addition, we considered only non-scheduled admissions, excluding scheduled surgical patients known to have lower hospital mortality rates [33].

We acknowledge several limitations. First, although multicentre, this is a retrospective study, intrinsically susceptible to have selection bias. Only critically ill metastatic cancer patients admitted to the ICU were included, so we do not have any information on in-ward or in emergency department triage decisions, by oncologists themselves or after multidisciplinary discussion including an intensivist. Moreover, unidentified confounding factors may have been overlooked in the multivariable analysis. Yet, biological and medical variables were collected prospectively with the information system. Second, we did not record any functional information from patients returned home, such as ECOG-PS or need for home care, for example. We supposed attending physicians would not discharge home bedridden or moribund patients. Third, in line with the previous limitation, we could not collect any data about the subsequent specific treatment and its feasibility. The ability to receive planned cancer treatment after ICU discharge is another relevant and meaningful endpoint in this population [34]. A prospective study would be needed to assess cancer treatment combined to quality-of-life indicators after ICU discharge.

## Conclusion

Despite underlying metastatic solid malignancies, more than one out of three (37.2%) patients returned home within 90 days after an unplanned admission to the ICU. Previous performance and nutritional status, ongoing specific treatment and low severity of the acute illness were found to be predictive for return home and survival at 1 year. These results should be utilised to inform meaningful conversations with clinicians, patients and family members to ensure appropriate decision making.

## Data Availability

The data sets used and analyzed during the current study are available from the corresponding author upon reasonable request.

## Abbreviations

ARF: Acute respiratory failure
BMI: Body mass index
CCI: Charlson comorbidity index
CI: Confidence interval
DFLST: Decisions to forgo life-sustaining therapies
ECOG-PS: Eastern cooperative oncology group performance status
HR: Hazard ratio
ICU: Intensive care unit
IMV: Invasive mechanical ventilation
IQR: Interquartile range
LDH: Lactate dehydrogenase
LOS: Length of stay
MV: Mechanical ventilation
NIV: Non-invasive ventilation
OR: Odds ratio
RRT: Renal replacement therapy
SAPS II: Simplified acute physiology score II
SOFA: Sequential organ failure assessment
